# Reduced serum Dickkopf-1 levels as a potential supportive biomarker for the development and severity of retinopathy of prematurity: a prospective observational clinical study

**DOI:** 10.3389/fped.2026.1917348

**Published:** 2026-09-28

**Authors:** Nuriye Emiroğlu, Hasan Çetin, Hüseyin Altunhan, Günhal Şatırtav, Saime Uygun

**Affiliations:** 1 Department of Pediatrics, Faculty of Medicine, Necmettin Erbakan University, Konya, Türkiye; 2 Department of Ophthalmology, Faculty of Medicine, Necmettin Erbakan University, Konya, Türkiye; 3 Department of Pediatrics, Selçuk University, Konya, Türkiye

**Keywords:** biomarker, dickkopf-1 (DKK-1), preterm infant, retinal neovascularization, retinopathy of prematurity (ROP), wnt/β-catenin signaling

## Abstract

**Introduction:**

Retinopathy of prematurity (ROP) involves impaired retinal vascular development, in which the Wnt/β-catenin signaling pathway plays a pivotal role in retinal vasculogenesis and angiogenesis. Dickkopf-1 (DKK-1) is a key endogenous inhibitor of this pathway. This study aimed to evaluate serum DKK-1 levels in preterm infants and explore its potential as a supportive biomarker associated with ROP development and severity.

**Methods:**

In this prospective observational clinical study, preterm infants with a gestational age ≤32 weeks or birth weight ≤1500 g were enrolled. Serum DKK-1 levels were measured via ELISA. ROP was classified according to the International Classification of Retinopathy of Prematurity (ICROP). Clinical characteristics and DKK-1 levels were compared between infants with ROP and a control group. Diagnostic performance was evaluated using Receiver Operating Characteristic (ROC) analysis, and independent risk factors were identified via multivariable logistic regression.

**Results:**

A total of 60 preterm infants were included, of whom 39 had ROP and 21 served as controls. Serum DKK-1 levels were significantly lower in infants with ROP compared to the control group (median 456.1pg/mL vs. 537.2pg/mL; *p* = 0.033). A stepwise decline in DKK-1 levels was observed as disease severity increased, with the lowest levels found in the treatment-requiring (TR) ROP subgroup. Although the overall comparison among the three groups did not reach statistical significance (Kruskal–Wallis, *p* = 0.066), a progressive decline in DKK-1 levels with increasing disease severity was observed. ROC analysis for TR-ROP vs. controls showed an area under the curve (AUC) of 0.679 (*p* = 0.041), with 76.2% sensitivity and 75.0% specificity at a cut-off of 480.41pg/mL. Multivariable logistic regression revealed that gestational age was the only independent predictor for ROP (*p* = 0.002), while DKK-1 levels showed a biological but not independent association in the model (*p* = 0.199).

**Conclusion:**

Reduced serum DKK-1 levels were associated with the presence and increasing severity of ROP. Although DKK-1 was not an independent predictor in multivariable analysis, these findings suggest that it may serve as a potential supportive biomarker for identifying infants at increased risk of severe ROP. Further large-scale, multicenter studies are needed to validate its clinical utility and to clarify its role in the Wnt/β-catenin signaling pathway.

## 1 Introduction

Retinopathy of prematurity (ROP) is a serious disease that occurs due to impaired retinal vascular development in preterm infants and can lead to blindness. Various molecular signaling pathways that regulate retinal vascular maturation play a role in the pathogenesis of ROP. Among these, the Wnt/β-catenin signaling pathway is critically involved in retinal vasculogenesis and angiogenesis (1–3).

Dickkopf-1 (DKK-1) is an endogenous inhibitor of the Wnt/β-catenin pathway and also acts as a regulator to maintain physiological levels of Wnt/β-catenin pathway activity (4). In other proliferative retinopathies, particularly diabetic retinopathy (DR), overactivation of this pathway has been shown to contribute to the progression of the disorder (1). Studies in patients with DR have demonstrated reduced serum DKK-1 levels, which were associated with retinal neovascularization and inflammatory processes (5–7). However, to our knowledge, there are no clinical studies that have specifically evaluated serum DKK-1 levels in ROP, especially in relation to different disease stages. The aim of this study was to explore serum DKK-1 levels in preterm infants to elucidate the potential role of DKK-1 in the pathogenesis and severity of ROP We further aimed to assess whether DKK-1 could serve as a predictive and guiding biomarker for ROP.

## 2 Materials and methods

### 2.1 Study design and participants

This prospective observational clinical study was conducted to evaluate the role of serum DKK-1 levels in the development of ROP among preterm infants with a gestational age (GA) ≤ 32 weeks and/or birth weight (BW) ≤ 1500 g. Eligible infants who were followed at the Neonatal Intensive Care Unit of Necmettin Erbakan University Medical Hospital between January 2022 and January 2024 were consecutively enrolled.

The study protocol was certificated by the institutional ethics committee, and written informed consent was obtained from all parents or valid custodian.

### 2.2 Clinical and perinatal data

Antenatal and postnatal variables potentially associated with ROP were recorded, including maternal age, antenatal corticosteroid use, preeclampsia/eclampsia, maternal diabetes, chorioamnionitis, *in vitro* fertilization, multiple gestation, mode of delivery, sex, GA, BW, small for gestational age (SGA; BW <10th percentile), need for resuscitation at birth, respiratory distress syndrome (RDS), surfactant therapy, duration of invasive and noninvasive ventilation and oxygen therapy, intraventricular hemorrhage (Papile grade > II), hemodynamically significant patent ductus arteriosus (PDA), early and late neonatal sepsis (clinical or culture-positive), necrotizing enterocolitis (NEC; modified Bell stage ≥ II), and bronchopulmonary dysplasia (BPD; oxygen requirement at 36 weeks postmenstrual age).

Exclusion criteria included infants with stage 2–3 hypoxic-ischemic encephalopathy, multiple congenital anomalies, severe congenital heart disease, or those who died during follow-up.

### 2.3 ROP screening and classification

The stage, zone, and presence of plus disease were classified according to the International Classification of Retinopathy of Prematurity (ICROP) (8). Screening examinations were initiated between postnatal weeks 4–6 and continued until complete retinal vascularization was achieved. Treatment criteria followed the Early Treatment for Retinopathy of Prematurity (ETROP) guidelines (9, 10). ROP was classified on a per-infant basis rather than per eye. When the severity differed between the two eyes, the eye with the more advanced stage was used to determine the ROP stage, zone, and treatment status. Consequently, each infant contributed only one observation to the statistical analyses, thereby avoiding inter-eye correlation.

### 2.4 Study endpoints

The primary endpoint was to compare serum DKK-1 levels between infants who developed ROP and those in the control group. The secondary endpoint was to evaluate DKK-1 levels among infants with ROP, comparing those who required treatment to those who did not.

### 2.5 Blood sampling and serum DKK-1 measurement

Blood samples were obtained at standardized time points to minimize variability. In infants with treatment-requiring ROP, samples were collected on the day of treatment. In infants with non–treatment-requiring ROP and in the control group, samples were obtained at 37 weeks postmenstrual age. Venous blood (3 mL) was collected from each infant. Samples were centrifuged at 4000 rpm for 5 min and stored at −80 °C until analysis.

To measure serum DKK-1 levels, a commercially available enzyme-linked immunosorbent assay (ELISA) kit was used according to the producer's guides.

### 2.6 Statistical analysis

All statistical analyses were performed using SPSS for Windows, version 18.0 (SPSS Inc., Chicago, IL, USA). Data were presented as number (n), percentage (%), mean ± standard deviation (SD), and median (interquartile range, IQR). The Mann–Whitney U test was used to compare non-normally distributed numerical variables between two groups. Comparisons among the three groups were performed using the Kruskal–Wallis test. No additional pairwise comparisons were performed in order to avoid inflation of type I error.

To identify independent risk factors for the development of ROP, a multivariable logistic regression analysis was performed using the ‘Enter’ method. Variables that showed a *p*-value of less than 0.20 in the univariate analysis, along with clinically significant parameters (Gestational Age, Preeclampsia, BPD, and DKK-1 levels), were included in the model. The model's goodness-of-fit was assessed using the Hosmer–Lemeshow test. Odds ratios (ORs) and 95% confidence intervals (CIs) were calculated to quantify the associations.

The diagnostic utility of serum DKK-1 levels for disease prediction was assessed using Receiver Operating Characteristic (ROC) curve analysis. Optimal cut-off values were determined based on the Youden index [maximum(sensitivity+specificity−1)]. For each cut-off, sensitivity, specificity, positive predictive value, and negative predictive value were calculated. Statistical significance was defined as *p* < 0.05.

### 2.7 *Post-hoc* power analysis

A *post-hoc* power analysis was conducted to determine the statistical strength of the study using G*Power software (version 3.1.9.7). Based on the observed serum DKK-1 levels, the effect size (Cohen's d) was calculated as 0.82. At a significance level (*α*) of 0.05, the achieved statistical power (1-β) was found to be 0.814. Because this analysis was performed after completion of data collection, the results should be interpreted cautiously and are considered supportive rather than confirmatory.

## 3 Results

### 3.1 Study population and ROP distribution

A total of 60 preterm infants were analyzed, highlighting a significant correlation between lower DKK-1 levels and the presence of ROP (*p* = 0.033). Of these, 39 (65%) developed retinopathy of prematurity (ROP), while 21 (35%) comprised the control group. Within the ROP group, 24 infants (61.5%) were classified as treatment-requiring and 15 (38.5%) as non–treatment-requiring.

### 3.2 Clinical characteristics

When comparing the ROP group with the control group, gestational age (*p* = 0.002), 5-min Apgar score (*p* = 0.021), and maternal preeclampsia (*p* = 0.037) were significantly lower in infants who developed ROP. No significant differences were observed between groups regarding birth weight, maternal age, sex, small for gestational age (SGA), respiratory distress syndrome (RDS), or bronchopulmonary dysplasia (BPD) (Tables 1, 2). Blood samples in the control and non–treatment-requiring ROP groups were obtained at 37 weeks' postmenstrual age, as described in the Methods. The mean postmenstrual age at blood sampling in infants with treatment-requiring ROP was 36.71 ± 2.45 weeks (95% CI: 35.60–37.83).

**Table 1:** Comparison of maternal, perinatal and demographic characteristics between ROP and control groups.

Characteristics	ROP Group (*N* = 39)	Control Group (*N* = 21)	p*
Maternal age (years)	27.0 (23.0–32.0) Min–Max: 17–38	29.0 (24.0–34.0) Min–Max: 19–39	0.397
Gestational age (weeks)	27.0 (26.0–29.0) Min–Max: 24–32	30.0 (28.0–31.0) Min–Max: 26–33	0.002
Birth weight (g)	960.0 (700.0–1,260.0) Min–Max: 570–1,775	1,030.0 (880.0–1,190.0) Min–Max: 600–1,600	0.556
APGAR score, 1st min	3 (3–4) Min–Max: 2–6	4 (3–5) Min–Max: 2–6	0.039
APGAR score, 5th min	4 (4–5) Min–Max: 3–7	5 (4–6) Min–Max: 3–7	0.021
Multiple pregnancy, *n* (%)	8 (20.5)	6 (28.6)	0.345
Sex, *n* (%)	Female: 22 (56.4) Male: 17 (43.6)	Female: 14 (66.7) Male: 7 (33.3)	0.439
Birth weight percentile, *n* (%)	AGA: 33 (84.6) SGA: 6 (15.4)	AGA: 14 (66.7) SGA: 7 (33.3)	0.101
Antenatal betamethasone, *n* (%)	None:8 (20.5) 1dose:9 (23.1) 2 doses: 22 (56.4)	None:3 (14.3) 1dose:9 (42.9) 2 doses: 9 (42.9)	0.279
Maternal hypertension/preeclampsia, *n* (%)	7 (17.9)	9 (42.9)	0.037
Gestational diabetes mellitus, N (%)	2 (5.1)	0 (0.0)	

Data are presented as median (IQR) or *n* (%). p: Mann–Whitney U test for continuous variables, Chi-square test for categorical variables. AGA, appropriate for gestational age; ROP, Retinopathy of prematurity; SGA, small for gestational age.

*p value.

**Table 2:** Comparison of neonatal morbidities and treatment characteristics between ROP and control groups.

Characteristics	ROP Group (*N* = 39)	Control Group (*N* = 21)	*p* *
Bronchopulmonary dysplasia, *n* (%)	16 (41.0)	10 (47.6)	0.623
Respiratory distress syndrome, *n* (%)	39 (100.0)	20 (95.2)	-
Hemodynamically significant PDA, *n* (%)	9 (23.1)	2 (9.5)	0.174
Necrotizing enterocolitis, *n* (%)	6 (15.4)	0 (0.0)	-
Proven sepsis, *n* (%)	6 (15.4)	3(14.3)	0.614
Asphyxia (Stage 1), *n* (%)	6 (15.4)	3 (14.3)	0.614
Intracranial hemorrhage, *n* (%)	3 (7.7)	2 (9.5)	0.578
Resuscitation at birth, *n* (%)	3 (7.7)	3 (14.3)	0.348
Surfactant therapy, *n* (%)	None:6 (15.4) 1dose:11 (28.2) 2 doses: 22 (56.4)	None:5 (23.8) 1dose:9 (42.9) 2 doses: 7 (33.3)	0.233
Non-invasive ventilation, *n* (%)	37 (94.9)	20 (95.2)	0.722
Invasive ventilation, *n* (%)	35 (89.7)	16 (76.2)	0.153

PDA, patent ductus arteriosus; ROP, retinopathy of prematurity.

* p:Chi-square test.

The ophthalmologic characteristics of infants with ROP are summarized in Table 3. Among the 39 infants with ROP, Zone II was the most common retinal location (69.2%), followed by Zone III (17.9%) and Zone I (12.8%). Among the 36 infants with stage information available, Stage 2 ROP was the most frequent (55.6%), followed by Stage 1 (27.8%) and Stage 3 (16.7%). Three additional infants were diagnosed with aggressive ROP (A-ROP). Plus disease was present in 22 infants (56.4%). Among the 24 infants with treatment-requiring ROP, 18 (75.0%) underwent laser photocoagulation and 6 (25.0%) received intravitreal anti-VEGF therapy.

**Table 3:** Ophthalmologic characteristics of infants with retinopathy of prematurity (*n* = 39).

Characteristic	*n* (%)
ROP Zone	
Zone I	5 (12.8)
Zone II	27 (69.2)
Zone III	7 (17.9)
ROP Stage^a^	
Stage 1	10 (27.8)
Stage 2	20 (55.6)
Stage 3	6 (16.7)
Plus disease	
Present	22 (56.4)
Absent	17 (43.6)
Treatment modality^b^	
Intravitreal anti-VEGF	6 (25.0)
Laser photocoagulation	18 (75.0)

ROP, retinopathy of prematurity; A-ROP, aggressive retinopathy of prematurity; anti- VEGF, anti- vascular endothelial growth factor.

a Stage information was available for 36 infants. Three infants were diagnosed with aggressive ROP (A-ROP), which is classified separately from the conventional staging system.

b Treatment modality is presented only for 24 infants with treatment-requiring ROP.

### 3.3 Serum DKK-1 levels

Plasma DKK-1 levels were significantly lower in infants with ROP compared to controls (median 456.1 pg/mL; IQR: 427.7–512.8 vs. 537.2 pg/mL; IQR: 466.2–595.9; *p* = 0.033) (Table 4).

**Table 4:** Comparison of DKK-1 levels between ROP and control groups.

Group	*n*	DKK-1 (pg/mL), Median (IQR)	*p* *
ROP (all patients)	39	456.1 (427.7–512.8)	0.033
Control	21	537.2 (466.2–595.9)	

* p: Mann–Whitney U test; IQR, interquartile range; DKK-1, Dickkopf-1; ROP, retinopathy of prematurity.

Within the ROP group, treatment-requiring infants had lower median DKK-1 levels than non–treatment-requiring infants (450.0 pg/mL; IQR: 422.0–496.6 vs. 488.5 pg/mL; IQR: 452.0–553.4). Although median DKK-1 levels showed a stepwise decrease from controls to non–treatment-requiring ROP and treatment-requiring ROP, the overall Kruskal–Wallis comparison did not reach statistical significance (*p* = 0.066). Nevertheless, the observed trend suggests a progressive decline in DKK-1 levels with increasing disease severity (Table 5; Figure 1).

**Table 5:** Comparison of serum DKK-1 levels among treatment-requiring ROP, non-treatment-requiring ROP, and control groups using the kruskal–wallis test.

Group	*n*	DKK-1 (pg/mL), Median (IQR)
ROP requiring treatment	24	450.0 (422.0–496.6)
ROP not requiring treatment	15	488.5 (452.0–553.4)
Control	21	537.2 (466.2–595.9)

Statistical comparisons: Overall comparison among the three groups (Kruskal–Wallis test): *p* = 0.066.

IQR, interquartile range; DKK-1, Dickkopf-1; ROP, retinopathy of prematurity.

**Figure 1:**
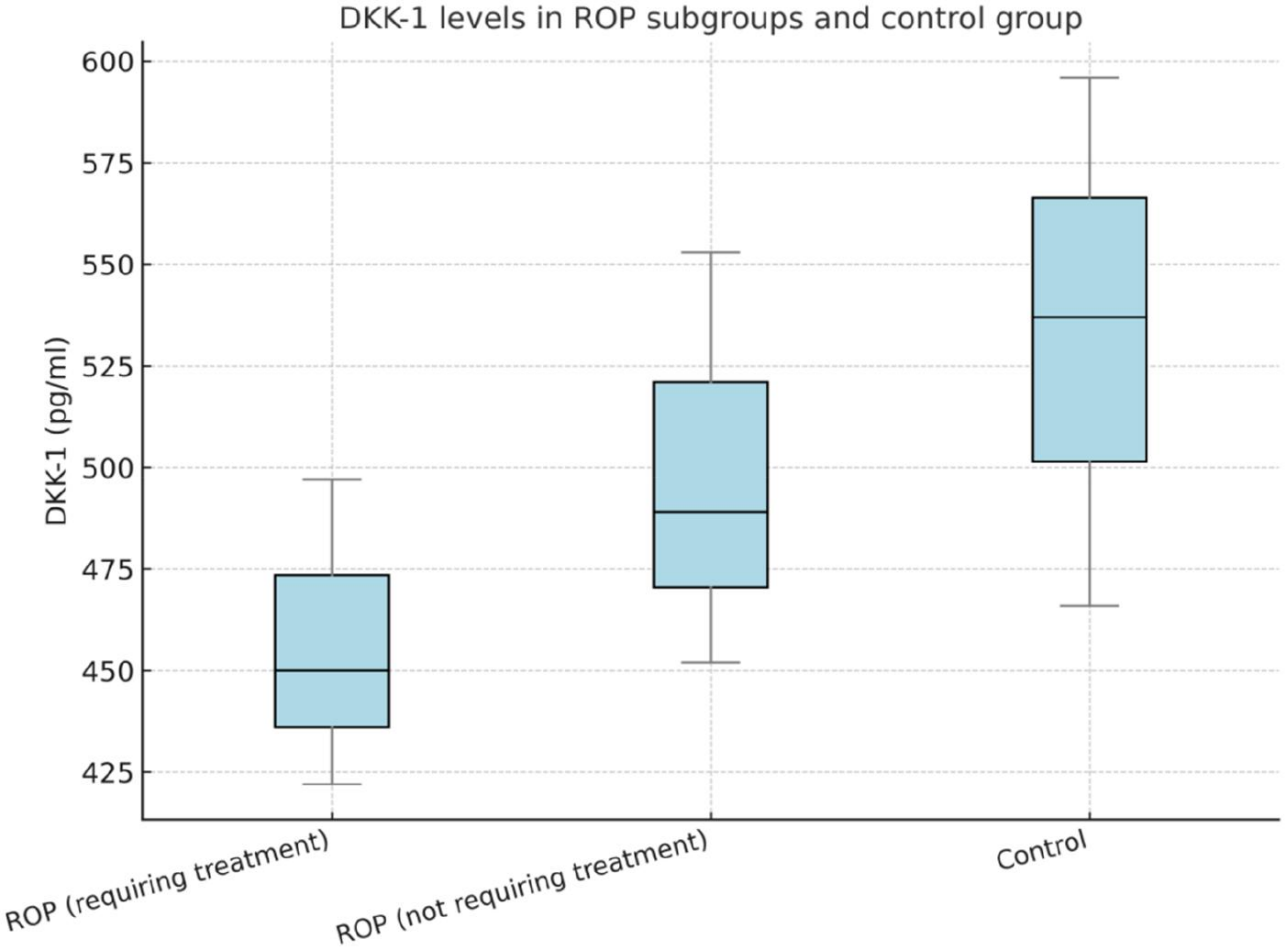
Distribution of serum dickkopf-1 (DKK-1) levels in treatment-requiring ROP, non-treatment-requiring ROP, and control groups. The box plots demonstrate a progressive decrease in median DKK-1 levels with increasing ROP severity. Although the overall comparison among the three groups did not reach statistical significance (Kruskal–Wallis test, *p* = 0.066), a stepwise downward trend in DKK-1 levels was observed. ROP, retinopathy of prematurity.

### 3.4 Diagnostic performance of DKK-1

Receiver Operating Characteristic (ROC) curve analysis was performed to assess the potential of DKK-1 as a biomarker for ROP. Optimal cut-off values were determined based on the Youden index [maximum(sensitivity+specificity−1)]. The cut-off values and corresponding diagnostic parameters are presented in Table 6 and figüre 2. For the Patient vs. Control comparison, the optimal cut-off value was 516.89 pg/mL, yielding a sensitivity of 66.7%, specificity of 76.9%, positive predictive value (PPV) of 60.9%, and negative predictive value (NPV) of 81.1%. For Treatment-Requiring ROP vs. Control, the cut-off was 480.41 pg/mL, with a sensitivity of 76.2%, specificity of 75.0%, PPV of 72.7%, and NPV of 78.3%. Our results indicate that DKK-1 could potentially function as a biomarker although they are not sufficient as a standalone diagnostic marker.

**Table 6:** Diagnostic performance of serum DKK-1 levels for retinopathy of prematurity (ROP) prediction using ROC curve analysis.

Comparison	AUC	*p*-value	Cut-off (pg/mL)	Sensitivity (%)	Specificity (%)	PPV (%)	NPV (%)
Patient vs. Control	0.668	**0**.**033**	516.89	66.7	76.9	60.9	81.1
Treatment-Requiring ROP vs. Control	0.679	**0**.**041**	480.41	76.2	75.0	72.7	78.3
Non-Treatment ROP vs. Control	0.651	0.147	516.89	66.7	76.9	60.9	81.1
Treatment-Requiring vs. Non-Treatment ROP	0.624	0.199	480.41	76.2	75.0	72.7	78.3

ROC, receiver operating characteristic; AUC, area under the curve; PPV, positive predictive value; NPV, negative predictive value; ROP, retinopathy of prematurity.

Bold values indicate the optimal cut-off values determined according to the Youden index.

### 3.5 Logistic regression analysis of factors associated with ROP

The multivariable logistic regression model was statistically significant (*χ*^2^ = 16.995; df = 4; *p* = 0.002), explaining 34.0% of the variance (Table 7). Gestational Age was the only independent predictor for ROP (*p* = 0.002; OR: 1.587; 95% CI: 1.178–2.139). Serum DKK-1 levels did not show a significant independent association in this model *p* = 0.199; OR: 1.003; 95% CI: 0.998–1.008).

**Table 7:** Multivariable logistic regression analysis of independent risk factors for ROP development.

Variable	*p*-value	Exp(B) (Odds Ratio)	95% Confidence Interval (Lower – Upper)
Gestational Age	**0**.**002**	1.587	1.178–2.139
Preeclampsia	0.227	2.398	0.580–9.921
BPD	0.199	2.529	0.615–10.402
DKK-1	0.199	1.003	0.998–1.008
Constant	0.001	0.000	-

BPD, bronchopulmonary dysplasia; CI, confidence interval; OR, odds ratio; DKK-1, Dickkopf-1, ROP, retinopathy of prematurity.

Bold values indicate statistically significant results (*p* < 0.05).

## 4 Discussion

DKK-1 is a natural inhibitor of the Wnt/β-catenin signaling pathway, which plays a critical role in cellular proliferation, differentiation, migration, and angiogenesis in many tissues, including the retina. Balanced activation of this pathway is necessary for normal retinal vasculogenesis, whereas dysregulated signaling can lead to pathological neovascularization and abnormal vessel development. β-catenin, as a key component of the pathway, contributes to retinal pigment epithelial cell proliferation (2, 6, 11–13). The biological significance of the reduction in DKK-1 levels observed in our study may lie in the loss of homeostatic control over Wnt signaling. DKK-1 acts as a molecular brake; therefore, its decline may remove the inhibition on the Wnt/β-catenin pathway, leading to the uncontrolled and pathological neovascularization characteristic of ROP (4).

In this study, serum DKK-1 levels were found to be significantly lower in infants diagnosed with ROP compared to preterm infants without retinopathy. This reduction was particularly pronounced in cases requiring treatment, suggesting a potential relationship between disease severity and DKK-1 levels. The significantly lower gestational age observed in infants with ROP is consistent with the established pathophysiology of the disease, as retinal vascular immaturity is a major determinant of ROP development. In our multivariable logistic regression analysis, gestational age remained the only independent predictor of ROP, highlighting the dominant contribution of prematurity to disease development. The clinical utility of serum DKK-1 is further underscored by our ROC analysis, which demonstrates a moderate but significant discriminative ability for ROP (AUC: 0.668–0.679). Crucially, the diagnostic performance was most robust in identifying treatment-requiring cases; a cut-off value of 480.41 pg/mL provided a sensitivity of 76.2% and a specificity of 75.0%. These findings suggest that while DKK-1 may not yet serve as a standalone definitive diagnostic tool, it offers substantial value as a supportive biomarker to help clinicians prioritize high-risk infants for closer monitoring or earlier intervention (Table 5; Figure 2).

**Figure 2:**
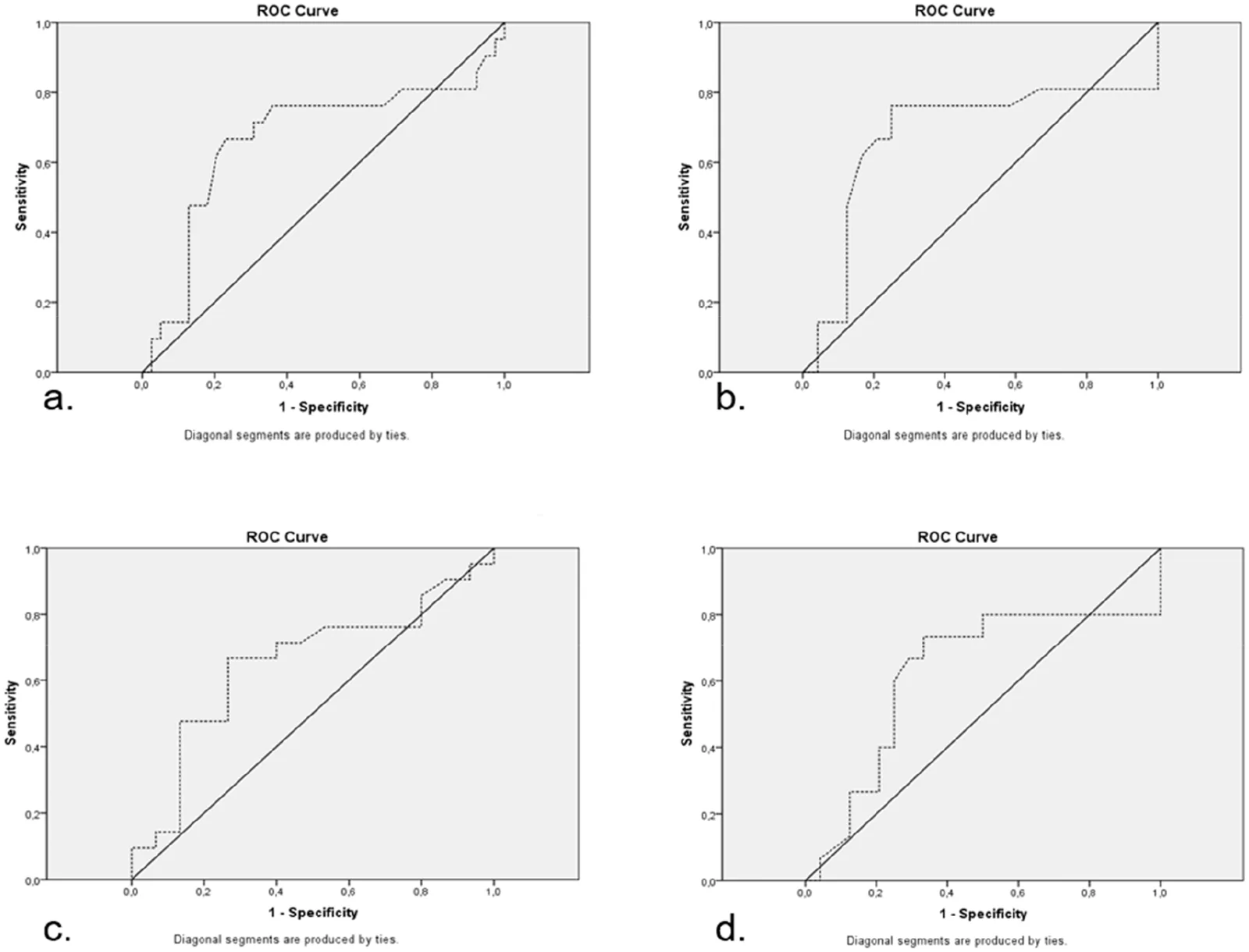
Receiver operating characteristic (ROC) curves of serum DKK-1 levels for predicting retinopathy of prematurity (ROP). AUC values for each comparison are as follows: **(a)** Patient vs. Control: AUC = 0.668 (*p* = 0.033). **(b)** Treatment-Requiring ROP vs. Control: AUC = 0.679 (*p* = 0.041). **(c)** Non-Treatment ROP vs. Control: AUC = 0.651 (*p* = 0.147). **(d)** Treatment-Requiring vs. Non-Treatment ROP: AUC = 0.624 (*p* = 0.199).

In our multivariable logistic regression analysis, while gestational age remained the strongest independent risk factor for ROP (*p* = 0.002), serum DKK-1 levels did not show an independent association (*p* = 0.199). This finding implies that DKK-1 levels are closely intertwined with the degree of prematurity itself. However, the lack of independent significance in the multivariable model does not diminish the biological importance of DKK-1 observed in our univariate and ROC analyses. The significant decline in DKK-1 levels in ROP cases—especially the stepwise decrease observed as severity increases—still points to its role as a potential clinical indicator of the pathological environment. The loss of statistical significance in the regression model may also be attributed to our relatively small sample size, which might lack the statistical power to separate the effect of DKK-1 from other strong clinical confounders like gestational age.

Several experimental studies have demonstrated the retinal tissue–protective and neovascularization-suppressing effects of DKK-1 (11, 14). For instance, Chen et al. reported that intravitreal administration of DKK-1 reduced inflammation, vascular leakage, and neovascularization in both human and animal models of diabetic retinopathy (11). Qiu et al. showed that plasma DKK-1 levels were lower in patients with diabetic retinopathy (DR) compared to both non-diabetic controls and diabetic individuals without retinopathy. Furthermore, DKK-1 levels were lower in proliferative DR (PDR) than in non-proliferative DR (NPDR) (5). These findings support the notion that reduced DKK-1 levels may be a common feature in proliferative retinal diseases. This biological mechanism is mirrored in other proliferative retinopathies. For instance, studies have shown that intravitreal administration of DKK-1 can reduce vascular leakage and inflammation in models of diabetic retinopathy. Our findings provide the first clinical evidence that a similar downregulation of DKK-1 occurs in ROP, suggesting that the protein's protective effect is diminished in severe cases (5).

Conversely, evidence from oxygen-induced retinopathy models shows that Wnt signaling components (Frizzled4, LRP5) are upregulated in neovascular areas, and pathological neovascularization is attenuated in cases of LRP5 or Dishevelled2 deficiency (1). Consistent with the literature, our findings suggest that decreased DKK-1 levels may be associated with ROP, particularly in severe, treatment-requiring cases. However, the difference between treatment-requiring and non-treatment-requiring groups did not reach statistical significance. Although the limited sample size is a constraint, our study achieved statistical significance in its primary goal of differentiating ROP cases from controls (*p* = 0.033). The observed stepwise decline in DKK-1 levels relative to disease severity provides a strong biological rationale for future, larger-scale validation studies.

Overall, our study supports the hypothesis that DKK-1 may act as a potential regulator or biomarker in ROP pathogenesis; ROC analysis highlights its potential utility in identifying infants at risk, although it is not sufficient as a definitive diagnostic tool. Beyond its role as a biomarker, DKK-1 represents a promising therapeutic target. If decreased DKK-1 levels contribute to the pathogenesis of ROP, stabilizing or augmenting DKK-1 levels through pharmacological intervention could potentially attenuate abnormal vessel growth. However, the diagnostic performance (AUC: 0.679 for treatment-requiring cases) suggests that DKK-1 should be integrated into a multi-marker screening approach rather than used as a standalone tool.

This study has several limitations. It was a single-center study with a relatively small sample size, and serum DKK-1 levels were measured at different time points. Another limitation is that the statistical power analysis was performed *post hoc* rather than during the study planning phase. Therefore, the calculated statistical power should be interpreted cautiously. Only DKK-1, not other components of the Wnt/β-catenin pathway, was evaluated, and potential confounding factors related to prematurity could not be fully controlled. Despite these limitations, our findings provide novel evidence that serum DKK-1 levels are associated with ROP development and may serve as a supportive biomarker. Future studies with larger sample sizes, longitudinal follow-up, and comprehensive analyses of other Wnt/β-catenin pathway components (e.g., Frizzled, LRP5/6, Dishevelled, TCF/LEF) are warranted to clarify the prognostic and therapeutic potential of DKK-1 in ROP.

## 5 Conclusion

In conclusion, reduced serum DKK-1 levels are significantly associated with both the occurrence and severity of ROP. Our study highlights DKK-1 as a novel supportive biomarker and a potential target for future therapeutic strategies aimed at modulating the Wnt/β-catenin pathway to prevent vision loss in premature infants. Future multicenter studies with larger cohorts are required to objectively evaluate the prognostic and therapeutic potential of DKK-1 in ROP.

## Data Availability

The raw data supporting the conclusions of this article will be made available by the authors, without undue reservation.
